# Sintilimab-induced photodistributed bullous pemphigoid: A case report

**DOI:** 10.1097/MD.0000000000041448

**Published:** 2025-02-07

**Authors:** Wenjuan Cui, Su Wang, Junzhu Xu, Xiaowei Shen, Murong Hu

**Affiliations:** a Department of Dermatology, Hangzhou Children’s Hospital, Hangzhou, Zhejiang, China; b Department of Dermatology, Hangzhou Third People’s Hospital, Hangzhou Third Hospital Affiliated to Zhejiang Chinese Medical University, Hangzhou, China; c Haining Maternity and Child Health Care Hospital, Jiaxing, China.

**Keywords:** bullous pemphigoid, PD-1/PD-L1 inhibitors, photodistribution, sintilimab

## Abstract

**Rationale:**

Immune-checkpoint inhibitors have emerged as a frontline treatment for a growing list of malignancies. Immunotherapy-induced bullous pemphigoid (BP) is a rare dermatological immune-related adverse event of immune-checkpoint inhibitor therapy immune-checkpoint inhibitor therapy. We report a case of immunotherapy-associated BP, with lesions presenting in a photodistribution. This case report aims to emphasize the early recognition of rare clinical manifestations induced by immunotherapy to improve patient prognosis.

**Patient concerns:**

The patient was a 77-year-old man with a history of right upper lung squamous cell carcinoma on sintilimab (anti-programmed cell death protein-1 [PD-1]) for over a year. After 12 months of initiation of PD-1 inhibitors, nonspecific cutaneous eruption appeared on his head, face, and extremities, mostly pruritic eczematous dermatitis with papules and plaques. The time to development of bullae after medication initiation was 16 months.

**Diagnoses:**

Sintilimab-induced BP.

**Intervention:**

Oral prednisone was gradually tapered to discontinuation following intravenous methylprednisolone; the skin lesions have basically recovered.

**Outcomes:**

Follow-up for 19 months showed no recurrence of the skin lesions.

**Lessons:**

This case report emphasizes that the clinical manifestations of BP induced by PD-1/programmed death ligand-1 inhibitors can be diverse. Dermatologists need to increase their awareness of BP caused by PD-1/programmed death ligand-1 inhibitors.

## 1. Introduction

Inhibitors of the programmed cell death protein-1 (PD-1; nivolumab and pembrolizumab) or its ligand, programmed death ligand-1 (PD-L1; durvalumab and atezolizumab), have emerged as a frontline treatment for a growing list of malignancies. Anti-PD-1/PD-L1 therapy frequently entails immune-related adverse events (AEs). Cutaneous immune-related AEs occur in 20% to 40% of patients treated with PD-1/PD-L1 inhibitors.^[1]^ The most common cutaneous complications are psoriasiform, eczematous, and lichenoid dermatoses.^[2]^ Bullous pemphigoid (BP) constitutes an uncommon dermatological immune-related AE of PD-1/PD-L1 inhibitors. In this article, we report a rare case of sintilimab-induced BP in a photodistribution.

## 2. Case presentation

**Figure 1.**
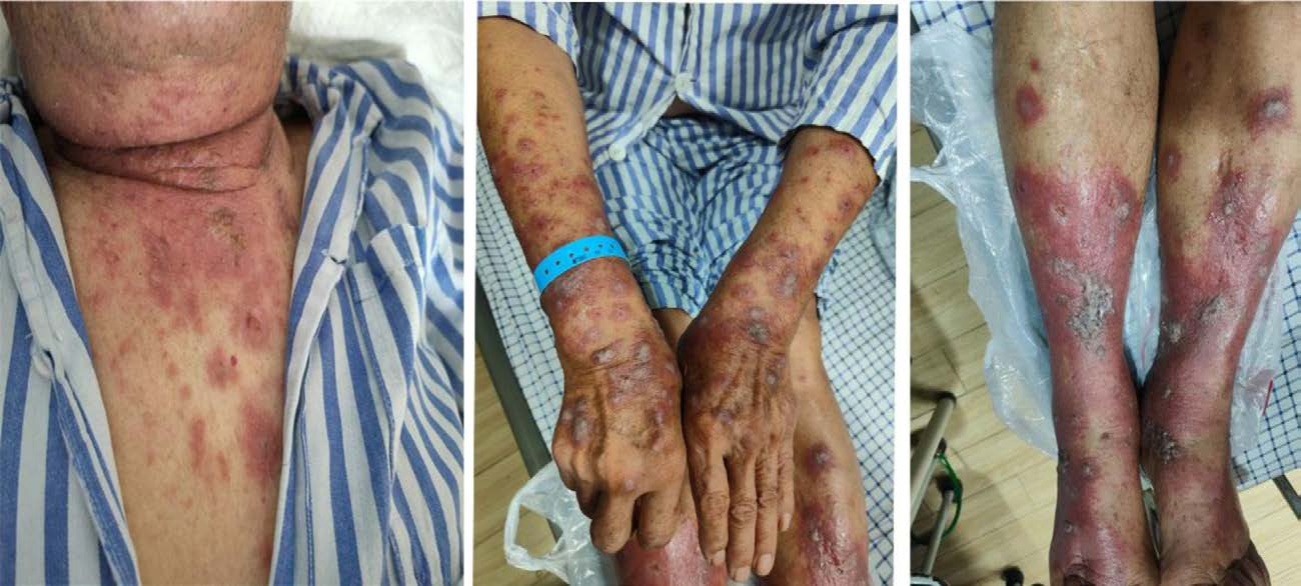
Patients before treatment (neck, chest, forearms, and lower limbs).

The patient is a 77-year-old male presenting with widespread pruritic erythematous papules and plaques on his head, face, and extremities for 4 months. Reviewing his medical history, he had a 16-month history of right upper lung squamous cell carcinoma and underwent chemotherapy with paclitaxel (200 mg) and carboplatin (500 mg) in combination with sintilimab (200 mg). Due to financial reasons, the patient only received 1 cycle of chemotherapy. The patient had a 10-year history of hypertension. No previous history of adverse drug reactions and drug-induced photosensitivity was reported. Physical examination showed the following: the skin on the face, neck, chest, forearms, and lower limbs shows patchy areas of thickened erythematous papules, plaques, and blisters (Fig. 1).

**Figure 2.**
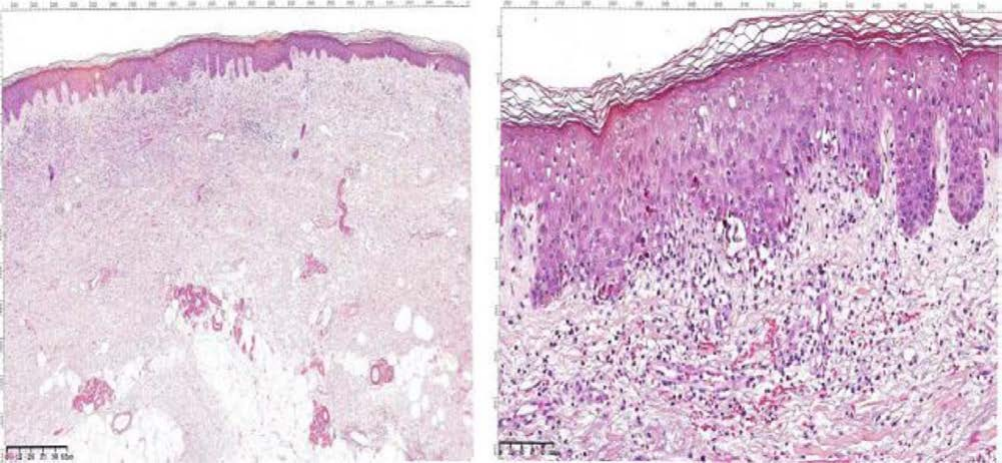
Histopathology showing mild epidermal hyperplasia with focal spongiotic edema (H&E stain, ◊40). Necrosis of keratinocytes in the upper layers of the basal layer, with mild perivascular infiltration of lymphocytes, histiocytes, and eosinophils in the superficial dermis, along with extravasation of red blood cells (H&E stain, ◊200).

**Figure 3.**
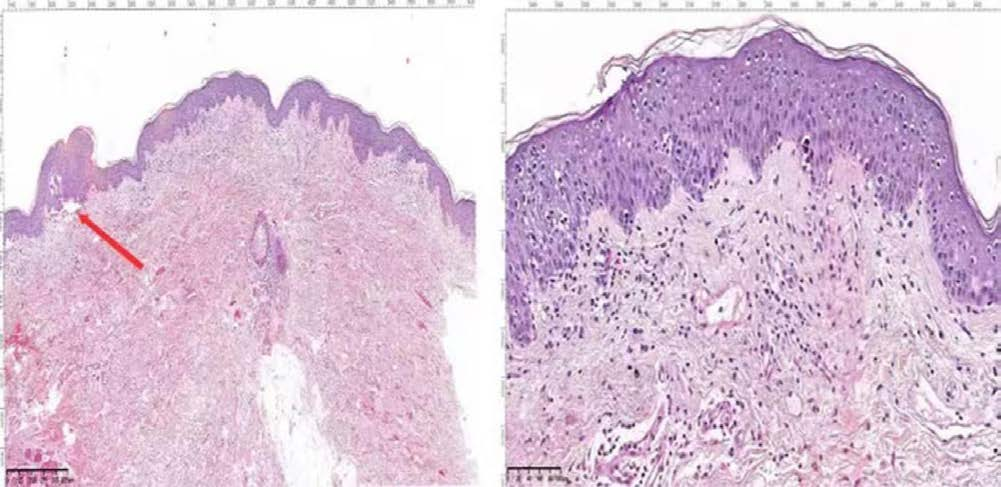
Histopathology showing subepidermal blister (H&E stain, ◊40). Superficial dermis infiltration of lymphocytes, some neutrophils, and few eosinophils (H&E stain, ◊200).

**Figure 4.**
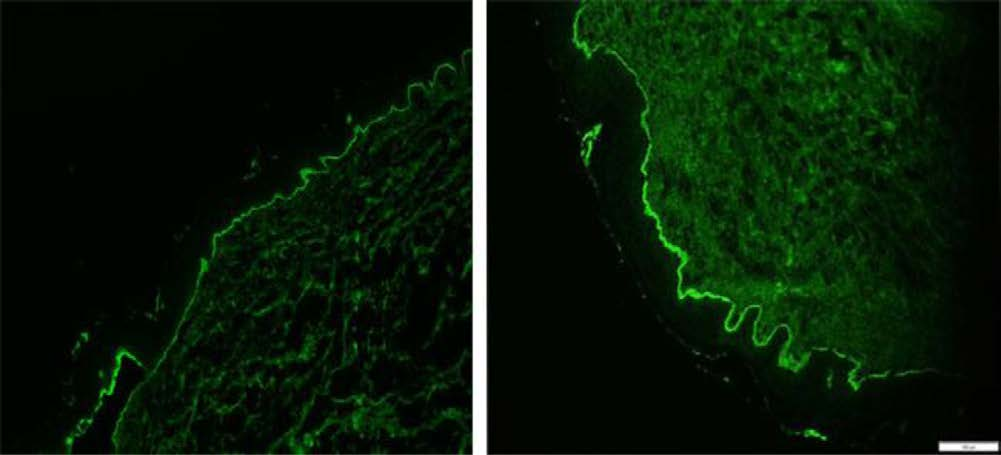
Direct immunofluorescence showed linear IgG and C3 along the basement membrane zone (◊100).

Laboratory findings showed the following: white blood cell count, 10.6 × 10^9^/L (reference range, 4–10 × 10^9^/L); C-reactive protein, 31.9 (reference range, 0–10) mg/L; erythrocyte sedimentation rate, 36 (reference range, 0–10) mm/h; and urinalysis, stool analysis, renal function tests, biochemical profiles, antinuclear antibodies, antineutrophil cytoplasmic antibodies, and thyroid function tests did not show any significant abnormalities. Abdominal ultrasound, chest X-ray, and electrocardiogram showed no significant abnormalities. Chest CT revealed scattered inflammatory lesions in both lungs. Pathological examination of the lower limb skin lesion is revealed (Fig. 2). Four days after the patient was hospitalized, scattered bullae appeared at the site of the primary rash, and pathological examination of the chest skin lesion is revealed (Fig. 3). Enzyme-linked immunosorbent assay studies were negative. Direct immunofluorescence is shown (Fig. 4). Given the clinical findings, his medication history, and histopathological features, the patient was diagnosed with sintilimab-induced BP.

## 3. Outcomes

After admission, the patient was treated with methylprednisolone sodium succinate of 60 mg qd and topical corticosteroids. The skin lesions gradually improved. On the 11th day of hospitalization, the treatment was changed to oral prednisone of 40 mg, and the patient was discharged. During follow-up visits at our outpatient clinic, prednisone was gradually tapered off and eventually discontinued after 19 months without recurrence of the rash.

## 4. Discussion

Dermatologic AEs are some of the most frequently observed toxicities of immune-checkpoint inhibitor therapy. Previous reports related to sintilimab (anti-PD-1) include pruritus, vitiligo, Stevens-Johnson syndrome, toxic epidermal necrolysis, BP, and so on.^[3]^ There have been reports of BP induced by PD-1 and PD-L1 inhibitors, but it is very unusual for BP to present in a photodistribution. Current literature reports that nivolumab (anti-PD-1) has photosensitivity, and all-grade incidence of photosensitivity was estimated at 1.5% (95% CI, 0.5%–4.4%).^[4,5]^

Because the patient’s lesions were predominantly localized in a sun-exposed location, and he has a documented history of long-term sun exposure, it is hypothesized that sintilimab may also be photosensitive. To our knowledge, there have been no previous reports of photosensitivity cases caused by sintilimab. Currently, there is only 1 reported case of anti-PD-L1–induced photodistributed BP,^[6]^ but the patient had no excessive sun exposure. Compared with the previous cases, the symptoms in this study’s patient are more severe, suggesting that photosensitivity may be involved in the pathogenesis. Possible mechanisms include that this photosensitivity might cause direct injury to the cutaneous basement membrane zone in sun-exposed areas, leading to antibody formation, or it could involve localized immune dysregulation. In addition, enzyme-linked immunosorbent assay studies are negative in these 2 cases, suggesting the presence of other antibodies, which provides new insights into the complex pathogenesis of this disease. Research indicates that bullae developed within 6 to 8 months of initiation of PD-1/PD-L1 inhibitors.^[7]^ In contrast to previous reports, in this patient, the bullae appeared 16 months after starting the medication.

Therefore, patients and clinicians should be informed of this potential occurrence and advised to avoid direct sunlight exposure and use appropriate photoprotection during treatment with sintilimab. However, this case report has limitations. This needs further research for confirmation.

## 5. Limitations

The limitations of this study include its focus on a single case, which limits generalizability to broader populations. While the 19-month follow-up showed a favorable prognosis and treatment discontinuation, the lack of long-term observation leaves uncertainty about the risk of recurrence. Further studies with larger cohorts and extended observation periods are needed to better understand the mechanisms and management of this condition.

## Author contributions

**Conceptualization:** Wenjuan Cui, Su Wang.

**Data curation:** Wenjuan Cui, Su Wang.

**Formal analysis:** Wenjuan Cui.

**Funding acquisition:** Wenjuan Cui.

**Investigation:** Wenjuan Cui.

**Methodology:** Wenjuan Cui.

**Project administration:** Wenjuan Cui.

**Resources:** Wenjuan Cui.

**Software:** Wenjuan Cui.

**Supervision:** Wenjuan Cui, Su Wang.

**Validation:** Wenjuan Cui, Junzhu Xu.

**Visualization:** Wenjuan Cui, Xiaowei Shen, Murong Hu.

**Writing – original draft:** Wenjuan Cui, Su Wang.

**Writing – review & editing:** Wenjuan Cui.
